# Supplementary figures and images for: Copper supplementation enhances pigmentation and induces dopamine production in ARPE19
Source: PLoS One. 2025 Jul 1;20(7):e0327352. doi: 10.1371/journal.pone.0327352 (PMC12212581; doi:10.1371/journal.pone.0327352)

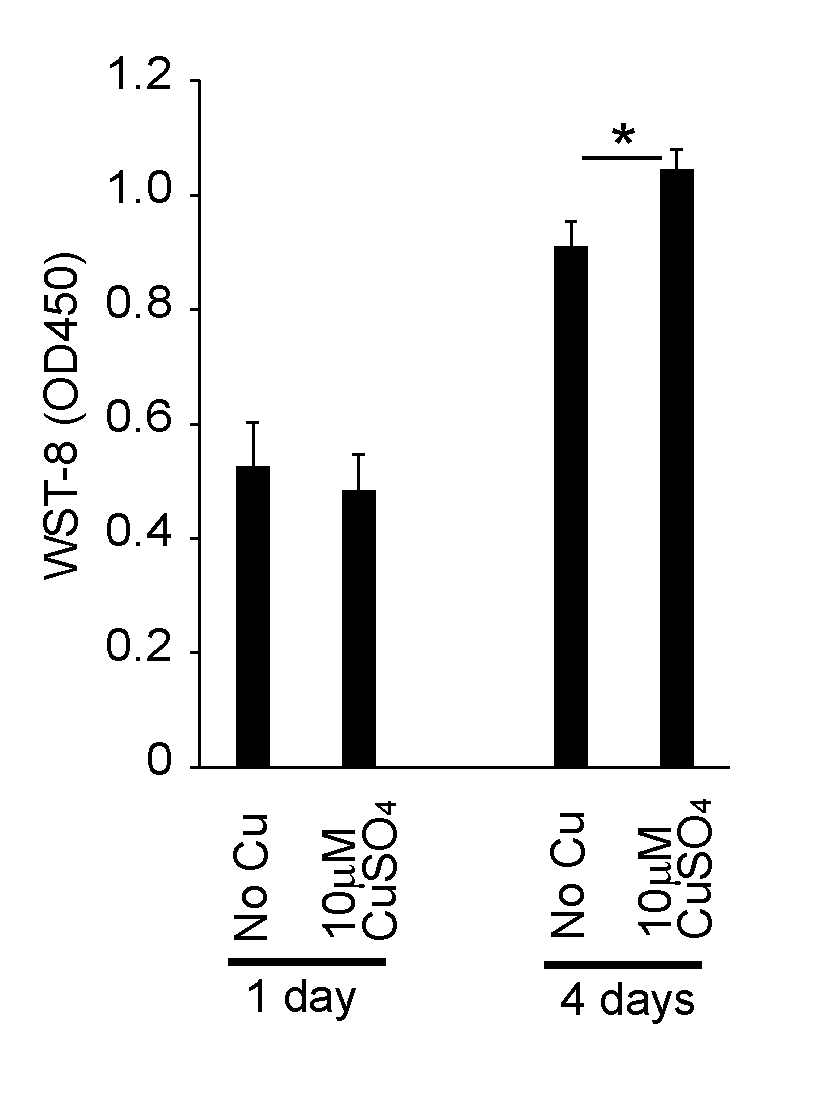

Supplement: S1 Fig — 1 day and 4 days with 10 μM copper sulfate did not show significant toxicity. * indicates p < 0.05 with Student’s t-test. (TIF) [file pone.0327352.s001.tif]

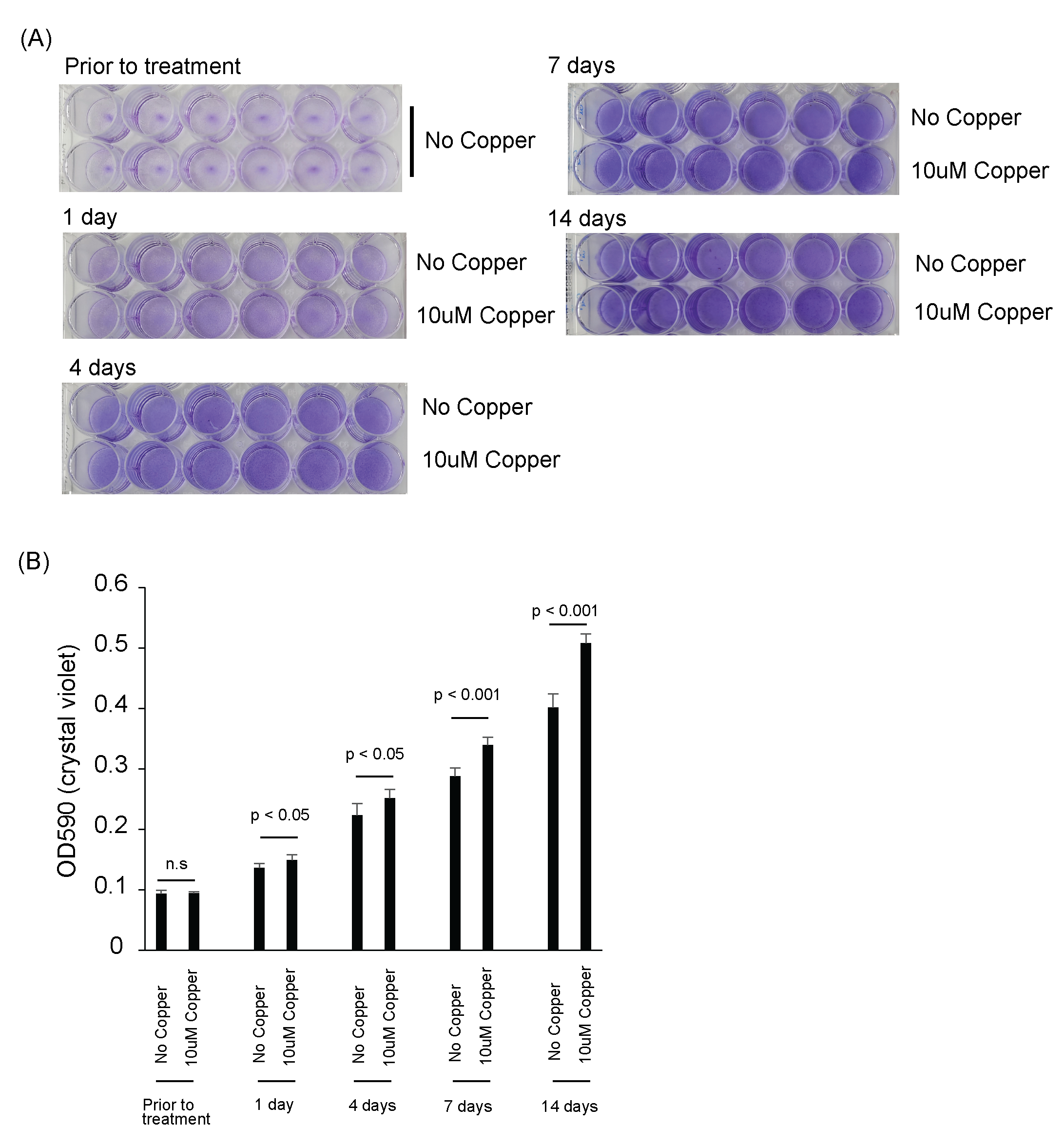

Supplement: S2 Fig — (A) Plate images after crystal violet staining. (B) After eluting the crystal violet, OD590 was measured. We found that copper treatment showed more cell growth rather than toxicity in our condition. N = 6. Student’s t-test was used for average comparison. (TIF) [file pone.0327352.s002.tif]

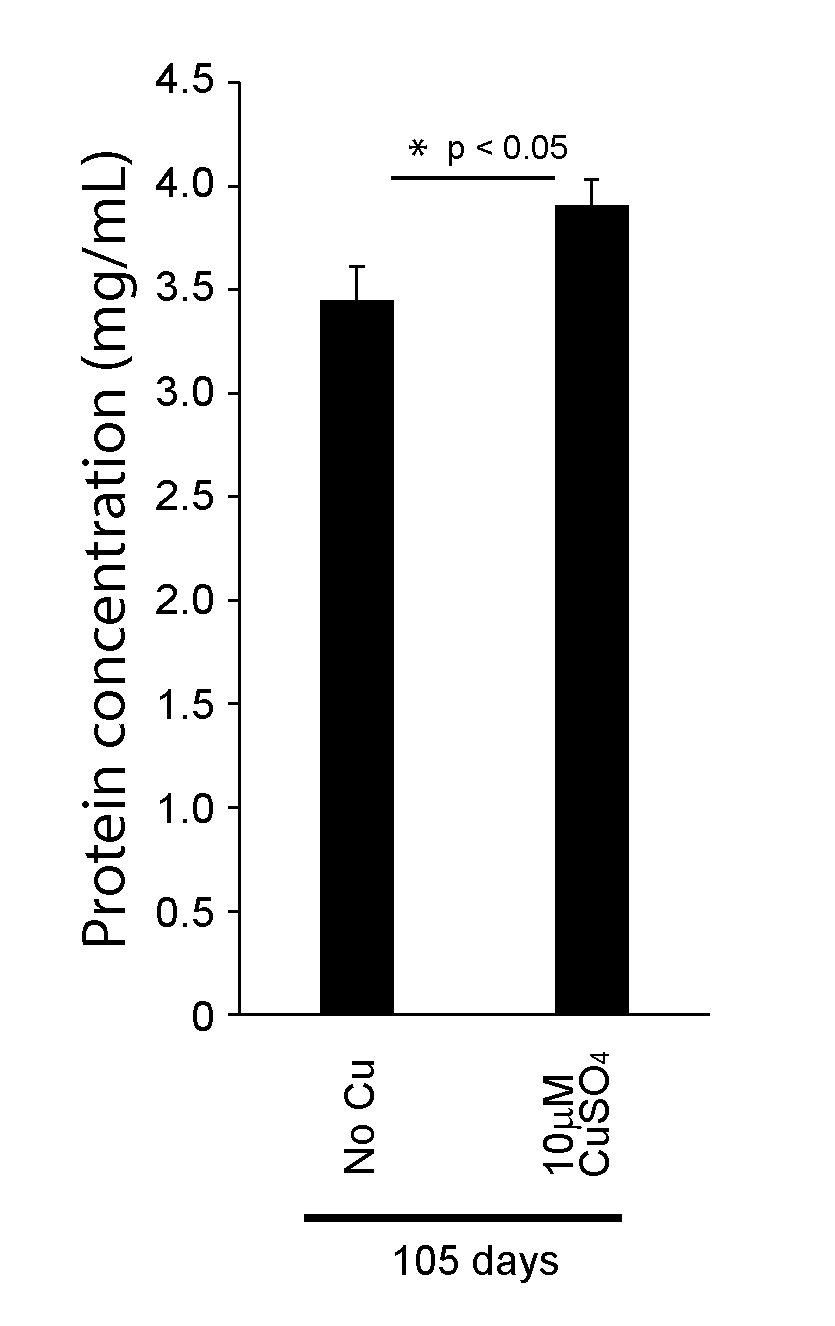

Supplement: S3 Fig — After 105 days culture, the cells were lysed with 250 μL RIPA buffer, and the protein concentration was measured by BCA assay. N = 3. * indicates p < 0.05 by Student’s t test. (TIF) [file pone.0327352.s003.tif]

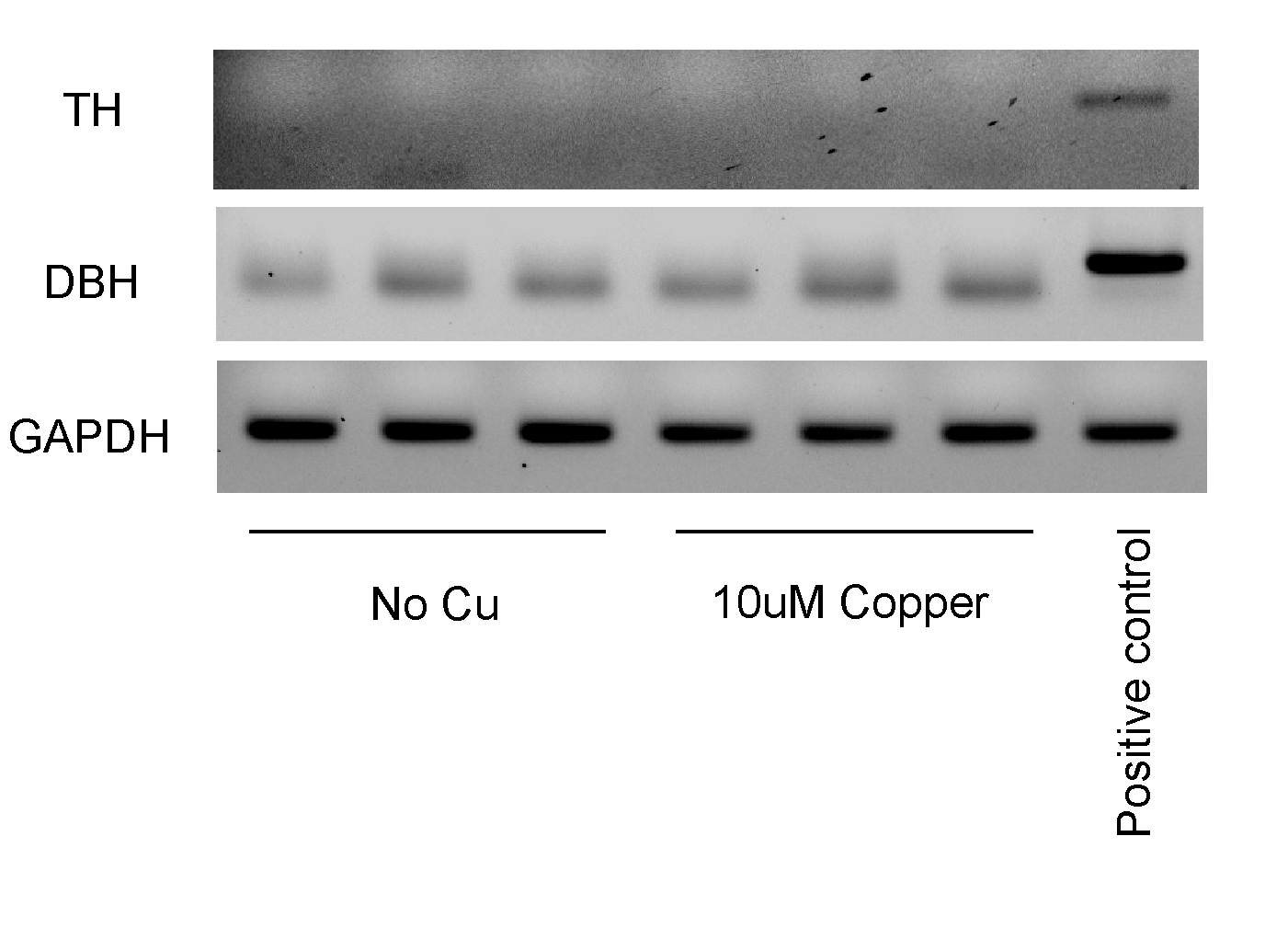

Supplement: S4 Fig — As a positive control, the same amount of human adrenal total RNA (Zyagen, San Diego, CA. Cat.no: HR-501) was used. (TIF) [file pone.0327352.s004.tif]
